# Comparative Study Regarding the Chemical Composition and Biological Activity of Pine (*Pinus nigra* and *P. sylvestris*) Bark Extracts

**DOI:** 10.3390/antiox10020327

**Published:** 2021-02-22

**Authors:** Adrian Nisca, Ruxandra Ștefănescu, Diana Ionela Stegăruș, Anca Delia Mare, Lenard Farczadi, Corneliu Tanase

**Affiliations:** 1 Doctoral School of Medicine and Pharmacy, “George Emil Palade” University of Medicine, Pharmacy, Sciences and Technology of Târgu Mureș, 38 Gheorghe Marinescu Street, 540139 Târgu Mureș, Mureș, Romania; 2 Department of Pharmacognosy and Phytotherapy, “George Emil Palade” University of Medicine, Pharmacy, Sciences and Technology of Târgu Mureș, 38 Gheorghe Marinescu Street, 540139 Târgu Mureș, Mureș, Romania; 3 National Research and Development Institute for Cryogenics and Isotopic Technologies—ICSI Ramnicu Valcea, 4th Uzinei Street, 240050 Ramnicu Valcea, Romania; diana.stegarus@icsi.ro; 4 Department of Microbiology, “George Emil Palade” University of Medicine, Pharmacy, Sciences and Technology of Târgu Mureș, 38 Gheorghe Marinescu Street, 540139 Târgu Mureș, Mureș, Romania; anca.mare@umfst.ro; 5 Chromatography and Mass Spectrometry Laboratory, Center for Advanced Medical and Pharmaceutical Research, “George Emil Palade” University of Medicine, Pharmacy, Sciences and Technology of Târgu Mureș, 38 Gheorghe Marinescu Street, 540139 Târgu Mureș, Mureș, Romania; lenard.farczadi@umfst.ro; 6 Department of Pharmaceutical Botany, “George Emil Palade” University of Medicine, Pharmacy, Sciences and Technology of Târgu Mureș, 38 Gheorghe Marinescu Street, 540139 Târgu Mureș, Mureș, Romania; corneliu.tanase@umfst.ro

**Keywords:** antioxidant, polyphenols, *Pinus sylvestris*, *Pinus nigra*, volatile compounds, catechin, antibacterial activity

## Abstract

The bark resulted after the industrial processing of wood represents a byproduct of the forestry industry, used in many suboptimal ways, being considered a natural waste. Currently, it has been highlighted that the bark of different woody plants may be an important source of several bioactive compounds, with various beneficial biological functions. The aim of this study is to evaluate and compare the chemical composition and biological activities of two different pine species (*Pinus nigra* and *Pinus sylvestris*) bark extracts. Ultrasound (UAE) and microwave (MAE) assisted extractions were performed in order to obtain the extracts. The total polyphenol (TPC) and total tannin (TTC) contents of the extracts were assessed via the Folin–Ciocâlteu method. The volatile and polyphenolic compounds were identified and quantified via a GC/MS analysis and an UPLC-PDA analysis, respectively. The antioxidant activity (AOA) was evaluated using the DPPH and ABTS assays, while the antibacterial activity was assessed using the minimum inhibitory concentration (MIC) protocol. The results showed that the *Pinus sylvestris* bark extracts obtained by UAE had a higher TPC, TTC and AOA, while the *Pinus nigra* bark extracts obtained by MAE had higher volatile compounds content (mainly α-pinene and β-pinene). Moreover, the inhibition of the bacterial growth was more efficient in the case of *Pinus sylvestris* extracts, Gram-positive bacteria being the most affected, while Gram-negative strains presented a relative resistance to the tested extract concentrations. These results may indicate the potential use of the pine bark extracts as antioxidant or antibacterial agents.

## 1. Introduction

In present days, the attention of researchers in the field is drawn towards the discovery of new bioactive compounds, which may be used for various purposes. A large group of such compounds is represented by the phenolics. These compounds are widely spread, providing important physiological and morphological traits for plants [1,2,3]. Although the main sources of phenolic compounds are fruits and vegetables, more recent studies have shown that the bark of woody vascular plants may represent a major source of phenolics [1,3]. Their role as protective agents against different plant pathogens might explain why these compounds are found in the bark of different species [3,4,5,6].

The effects of these compounds on human health are also intensely studied and most often linked to their antioxidant activity. Due to antioxidant activity polyphenols may prevent the development of different disorders such as neurodegenerative, respiratory and cardiovascular diseases, diabetes or arthritis [7,8,9,10]. Moreover, phenolic compounds have antimicrobial effects against different bacteria, exhibiting synergistic effects when they are combined with standard antibiotics [11,12,13,14,15]. Antimycotic effects against different species of the *Candida*, *Penicillium* and *Aspergillus* [16,17,18], and antiviral activities against HIV and *Herpes simplex* [19,20,21] were also highlighted in studies regarding phenolic compounds. Additionally, antitumoral effects against different malignant cell lines and the potential synergy with classic cancer drugs, were both revealed in previous studies [1,3,22].

*Pinus sylvestris* L. (scots pine) and *Pinus nigra* L. (black pine) are both species of the Pinaceae family. *Pinus sylvestris* is a common pine species, intensely studied, thus the phytochemical composition of its bark is well known, phenolic compounds like myricetin, eleutheroside, quercetin [23], vanillic acid, catechin, ferulic acid and taxifolin [24] being identified. Volatile compounds like α-pinene and β-pinene were also found in bark extractives of *Pinus sylvestris* [23]. These compounds might be the reason for some of the bark extract effects (i.e., anti-inflammatory via NO inhibition) [24,25]; antiproliferative against prostate cancer cells [26]; suppression of adipocyte differentiation and pancreatic lipase inhibition [27]. In contrast, data about *Pinus nigra* bark extracts is scarce, thus the potential health benefits of these extracts are not well known. However, the antibacterial [28], antioxidant and antiproliferative activities of some *Pinus nigra* bark extracts were previously revealed. These effects might be caused by the presence of catechin (monomers, dimers and trimers), ferulic acid esters of catechin and taxifolin [26].

Data about the toxicity profile of these specific pine species extracts could not be found in the literature, but studies conducted on other commercially available pine bark polyphenolic extracts were regarded as safe for topical and internal use, with no alarming side effects (even at high doses) being presented [29].

Therefore, the aim of this study was to present a comparative evaluation of the chemical composition and biological activity of some *Pinus sylvestris* and *Pinus nigra* bark extracts. This aim was achieved by determining the total (TPC) and individual polyphenol content, the total tannin content (TTC), the volatile compounds content of the bark extracts and their antioxidant activity (AOA) and antibacterial activity (against different bacterial strains).

## 2. Materials and Methods

### 2.1. Plant Samples and Bacterial Strains

The *P. sylvestris* and *P. nigra* barks used for extraction were collected in March from different locations in Romania. *P. sylvestris* samples were collected from Vama (47°34′19″ N, 25°42′55″ E), Suceava county, Reghin (46°47′04″ N, 24°41′20″ E), Mureș county and Mediaș (46°10′21″ N, 24°19′37″ E), Sibiu county. The *P. nigra* sample was collected from Dorohoi (47°56′53″ N, 26°23′12″ E), Botoșani county. These samples were collected by using the itinerary method. At each location, 3–5 trees of each species were randomly selected. The ages of the trees varied between 20 and 35 years. The bark samples were taken at 1–1.5 m of stem height. Afterwards the vegetal material was ground with GRINDOMIX GM 200 mill (Retsch GmbH, Haan, Germany).

The bacterial strains used in this study, *Staphylococcus aureus* (ATCC 25923), methicillin resistant *Staphylococcus aureus* (ATCC 43300), *Escherichia coli* (ATCC 25922), *Klebsiella pneumoniae* (ATCC 700603) and *Pseudomonas aeruginosa* (ATCC 27853), were provided by the Microbiology Department from the “G.E. Palade” University of Medicine, Pharmacy, Sciences and Technology from Târgu-Mureș.

### 2.2. Chemicals and Reagents

The reagents used for the extraction procedures, total polyphenolic and tannins content determination and for the antioxidant activity assays were: ethanol 96% (*v*/*v*) used to prepare the 50% and 70% solutions and Na_2_CO_3_, purchased from Chemical Company SA, Iași, Romania; gallic acid, 2,2-diphenyl-1-picrylhydrazyl (DPPH), 2,2’-azino-bis(3-ethylbenzothiazoline-6-sulfonic acid) (ABTS), hide powder and pyrogallol acquired from Sigma-Aldrich Chemie GmbH, Steinheim, Germany and the Folin–Ciocâlteu reagent obtained from Merck KGaA, Darmstadt, Germany.

The chemical standards (α-pinene, β-pinene, 3-carene, limonene, camphene, α-phellandrene, sabinene, mircene, tryciclene, gallic acid monohydrate, eleutheroside B, catechin, epicatechin, vanillic acid, sinapic acid, taxifolin and quercetin) used to identify the volatile and phenolic compounds in the extracts were acquired from Sigma-Aldrich Chemie GmbH, Steinheim, Germany.

Eleven standards were used for the UPLC-PDA analysis: gallic acid, vanillic acid, sinapic acid, chlorogenic acid, quercetin, eleutheroside B, taxifolin, luteolin-7-O-glucoside, luteolin-3′,7-diglucoside, epicatechin and catechin. Stock solutions for each standard were prepared in methanol. Calibration curves were established on 6 data points covering a concentration range of 5–50 µg/mL.

### 2.3. Extraction

The extracts were obtained by using two different extraction methods: ultrasound assisted extraction (UAE) and microwave-assisted extraction (MAE).

For the UAE 1 g of pine bark was treated with 20 mL of 70% ethanol solution in a volumetric flask. This mixture was left in an Elma Transsonics ultrasound bath (Elma Schmidbauer GmbH, Singen, Germany) for 30 min at 65 °C and then filtered through filter paper. The filtered extracts were brought into 20 mL volumetric flasks and completed with the 70% ethanol solution [30].

For the MAE 2 g of pine bark were treated with 20 mL of 50% ethanol solution in a volumetric flask. The mixture was microwaved with a power of 300 W for 4 min and then filtered through filter paper. The filtered extract was then brought into a 20 mL volumetric flask and completed with the 50% ethanol solution [31].

The result of the extraction processes was the following experimental variants: PSV US. *P. sylvestris* (Vama) extract acquired by UAE; PSR US. *P. sylvestris* (Reghin) extract acquired by UAE; PSM US. *P. sylvestris* (Mediaș) extract acquired by UAE; PND US. *P. nigra* (Dorohoi) extract acquired by UAE; PSV M. *P. sylvestris* (Vama) extract acquired by MAE; PSR M. *P. sylvestris* (Reghin) extract acquired by MAE; PSM M. *P. sylvestris* (Mediaș) extract acquired by MAE and PND M. *P. nigra* (Dorohoi) extract acquired by MAE. A duplicate extraction was performed for each method, resulting in 2 similar extracts for each experimental variant. In the following assays 3 measurements were performed for each one of these extracts, thus each assay consisted in 6 separate measurements for every variant.

### 2.4. Total Polyphenol Content (TPC)

The total polyphenolic content was measured by the Folin–Ciocâlteu method [32] and using the gallic acid as a reference standard. Brought in a vial were 40 μL of extract (diluted 1:5), 3160 μL distilled water, 200 μL of Folin–Ciocâlteu reagent and 600 μL of 20% Na_2_CO_3_ solution. The mixture was shaken and then left in a dark room at room temperature for 30 min. Afterwards, the absorbance of the solution was measured at 765 nm in a Specord 210 UV–Vis spectrophotometer (Analytik Jena AG, Jena, Germany). The TPC was expressed as gallic acid equivalents per gram of vegetal material (mg of GAE/g vegetal material). Six measurements were performed for each variant. A similar method was described previously [1].

### 2.5. Total Tannin Content (TTC)

The TTC was determined by dosing the polyphenols, which were adsorbed by the hide powder. This method is described in the European Pharmacopeia 7th Edition [33].

Briefly, for the determination of total polyphenols, 2 mL of diluted extract (1:25) were treated with 1 mL of Folin–Ciocâlteu reagent and 10 mL of distilled water. The mixture was then completed to 25 mL in a volumetric flask with a 29% Na_2_CO_3_ solution. The flask was shaken and left in a dark room at room temperature for 30 min. Afterwards the absorbance of the solution was measured at 760 nm (A_1_). Six measurements were performed for each variant.

To determine the content of polyphenols, which were not adsorbed on the hide powder, 10 mL of diluted extract were brought into a volumetric flask with 0.1 g of hide powder. The mixture was shaken for 60 min and then filtered. Of filtered solution 2 mL were then treated, as previously described, for the total polyphenols determination (A_2_). Six measurements were performed for each variant.

The TTC was determined by comparing the absorbance difference (A_1_ − A_2_) to a pyrogallol solution representing an external standard. The external standard solution was prepared by dissolving 50 mg of pyrogallol in 100 mL distilled water and then applying a 20-fold dilution. The final TTC was expressed as mg of pyrogallol/g vegetal material.

### 2.6. Analysis of Volatile Compounds

The main volatile compounds contained by the extracts were analyzed by gas chromatography in a Varian 450-GC with a FID detector and TG-WAXMS capillary column (60 m × 0.32 mm ID × 0.25 µm film thickness). The extracts attained via the extraction methods described above were filtered through a 0.5 µm microporous polytetrafluoroethylene filter and then 1 µL of the filtered extract were directly injected with a split ratio of 1:10. Three injections were performed for each variant. The carrier gas was helium (He) at a flow rate of 1.2 mL/min. The column temperature program used for the volatile compound separation was from 40 (held for 2 min) to 90 °C with a 5 °C/min rate, then up to 120 °C (held for 2 min) with a 10 °C/min rate and lastly up to 180 °C (held for 4 min). The injector and detector temperatures were set to 240 °C and 270 °C. The identification of the target compounds was performed by comparing the retention times of the individual compounds in a chromatogram recorded on a standard solution with the retention times of the same compounds in the chromatograms recorded for the extracted samples. The samples and standards were properly diluted before the injection. Qualitative and quantitative data was collected after this analysis.

### 2.7. UPLC-PDA Analysis

UPLC analysis of the polyphenolic compounds was performed according to a previous described method [34]. For this analysis, an UPLC Flexar FX-10 Perkin Elmer system was used, consisting of a binary pump, inline degaser, autosampler, column thermostat and a Flexar FX-PDA UHPLC detector. All solvents were LC–MS/MS grade and the reagents were of the highest available purity. Aliquots of each sample were injected into a Luna C18 (2) column (3 µm particle size, 150 mm × 4.6 mm) at a flow rate of 1 mL/min. The elution was performed with a 0.1% formic acid (phase A)/acetonitrile (phase B) gradient. The monitoring wavelengths were: 210, 280 and 370. The identification of compounds was carried out by comparing the retention time of the peaks and their UV–Vis spectra with that of the reference standards. All extracts were filtered prior to analysis through a 0.45 µm microporous cellulose syringe filter.

### 2.8. Antioxidant Activity (AOA) Assays

#### 2.8.1. DPPH Assay

For the AOA evaluation by DPPH method a 10-fold dilution was performed for PSM US, PND US, PSR US, PSV US, PSM M and PND M variants and a 20-fold dilution for the PSV M and PSR M variants. Of each sample 100 µL were treated with a 2.5 mL 0.1 mM DPPH solution. The mixture was shaken and then left in a dark room at room temperature for 30 min. The sample and DPPH solution absorbance were measured at 517 nm. Six measurements were performed for each variant. The inhibition percentage was calculated using the following formula [35]:

inhib % = (A_0_ − A_1_)/A_0_*100 (1)

A_0_—DPPH solution absorbance and A_1_—sample absorbance

#### 2.8.2. ABTS Assay

For the AOA evaluation by ABTS method, a procedure described previously with slight modifications, was used [36]. Briefly, this method consists in the treatment of 100 µL of tested sample with 100 µL of ABTS reagent. The mixture is shaken and left in a dark room for 5–6 min. For the measurement of sample and ABTS solution absorbance at 734 nm, an Epoch microplate reader (BioTek Instruments Inc., Winooski, USA) was used. Six measurements were performed for each variant. The inhibition percentage was determined with the formula mentioned at the DPPH method.

### 2.9. Antibacterial Activity

The antibacterial activity was evaluated by assessing the minimum inhibitory concentration (MIC), which represents the lowest extract concentration needed to inhibit the bacterial growth and reproduction. In order to establish MIC, the microdilution method was used. The protocol was previously described in a study conducted by Tanase C. et al. [37]. In addition, 3 µL of 0.015% resazurin solution were added in each well, in order to facilitate the result interpretation. The microplate was incubated again at 37 °C for 2–4 h. The MIC was considered the last well (dilution) in which resazurin did not change its color from purple to pink (bacterial growth was inhibited) [38]. To assess only the antibacterial activity of the phytochemicals from the extracts, the alcohol used for extraction was evaporated before testing.

### 2.10. Statistical Analysis

The statistical analysis was done in GraphPad Prism 7, using a unidirectional ANOVA test to determine the difference of variances between the analyzed data series. If a difference occurred, then a Tukey test was used. This test is often used to compare the difference between multiple data series means and their significance (*p* ≤ 0.05). These tests were used to compare the differences between the extracts’ TPC and TC, AOA and volatile compounds content.

## 3. Results and Discussions

### 3.1. Total Polyphenol Content (TPC)

The polyphenol content of the alcoholic extracts was determined spectrophotometrically, using the Folin–Ciocâlteu method. TPC was calculated using the absorbance of each sample at 765 nm, also considering the gallic acid standard solution calibration curve: y = 31.07x + 0.1028. The results were expressed as mg GAE/g vegetal material.

**Figure 1:**
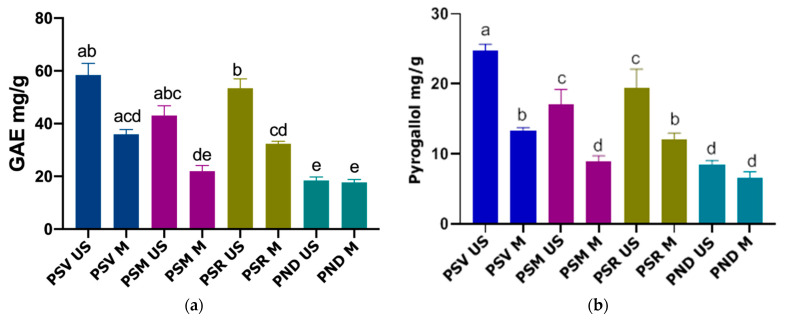
(**a**) Total polyphenol content (TPC) of the pine species bark extracts; (**b**) total tannin content (TTC) of the pine species bark extracts; different letters indicate significant differences; PSV US—*P. sylvestris* (Vama) extract obtained by UAE; PSV M—*P. sylvestris* (Vama) extract obtained by MAE; PSM US—*P. sylvestris* (Mediaș) extract obtained by UAE; PSM M—*P. sylvestris* (Mediaș) extract obtained by MAE; PSR US—*P. sylvestris* (Reghin) extract obtained by UAE; PSR M—*P. sylvestris* (Reghin) extract obtained by MAE; PND US—*P. nigra* (Dorohoi) extract obtained by UAE; PND M—*P. nigra* (Dorohoi) extract obtained by MAE.

Figure 1a shows that the PSV US variant (58.41 ± 10.9 mg/g) has the highest absolute value of TPC, followed by the PSR US (53.41 ± 8.74 mg/g) and PSM US (43.07 ± 9.03 mg/g) variants. The differences between these variants had no statistical significance. In comparison with the extracts obtained by MAE, PSV US showed a significantly higher TPC, excepting PSV M. By comparing PSV US to the other extracts obtained by UAE, it can be observed that only PND US had a significantly lower TPC. PSM US differed significantly from the same experimental variants as PSV US, having a higher TPC, except for PSR M (32.34 ± 2.38 mg/g). Between PSM US and PSR M a significant difference could not be found.

Although PSV US had the highest absolute value of TPC, PSR US had a significantly higher TPC compared to all the other extracts, excluding PSV US and PSM US.

Lastly, it was observed that the PND US variant (18.46 ± 3.2 mg/g) contained significantly lower amounts of polyphenols compared to all the other tested extracts, excepting PND M, which had a lower TPC, but the difference was not statistically significant.

Comparing our results to the results of a study conducted by Skrypnik L et al. on *P. sylvestris* extracts obtained by hot water extraction, it can be observed that our *P. sylvestris* extract with the minimum TPC (PSM M) contained a higher amount of polyphenols (22.00 ± 5.11 mg/g) than the values presented in the baseline study (12.33 ± 1.48 mg/g). In the baseline study TPC was also expressed in mg GAE/g vegetal material [39]. However, the comparison between our *P. nigra* extracts and the aqueous and acetonic extracts obtained by Bianchi S et al. from *P. nigra* bark, shows that the experimental variants (PND US; PND M) were inferior regarding the TPC. The TPCs found in the baseline study were 28.6 ± 2.1 mg/g (aqueous extracts) and 38.8 ± 1.2 mg/g (acetonic extracts) compared to 18.46 ± 3.2 mg/g in the PND US extract. In this case highly purified condensed tannins were used for calibration [40].

### 3.2. Total Tannin Content (TTC)

To assess the extracts’ tannin content, a dosing method using the Folin–Ciocâlteu reagent was used. The tannin contents per 1 g of vegetal material are presented in Figure 1b. These results were expressed as mg pyrogallol/g of vegetal material.

It can be observed that PSV US, PSR US and PSM US had the highest TTC out of all the experimental variants. However, PSV US (24.74 ± 0.95 mg/g) had significantly higher quantities of tannins compared to all extracts, as shown by the statistical analysis. Although PSR US (19.37 ± 2.76 mg/g) had shown a higher TTC than PSM US (17.07 ± 2.16 mg/g), this difference had no statistical significance. However, both of these variants had significantly higher TTC than almost all of the other extracts, excepting PSV US. Out of all the extracts obtained by UAE, PND US (8.50 ± 0.55 mg/g) presented the lowest TTC, having a higher content only compared to the analogue variant obtained by MAE (PND M), but the difference was not significant. It can be seen that the extracts obtained by MAE contained lower levels of tannins, PSV M (13.32 ± 0.47 mg/g) being the richest extract in these compounds. A significantly higher TTC was registered compared to PSM M and PND M, but not compared to PSR M.

These results were compared to the results of a study conducted on *P. sylvestris* bark aqueous extracts and it can be observed that even the experimental variant with the lowest TTC, out of the extracts obtained from *P. sylvestris* (PSM M), had greater amounts of tannins (8.93 ± 0.81 mg/g) compared to the extracts obtained in the baseline study (8.46 ± 1.35 mg/g). The baseline study expressed the TTC as mg GAE/g vegetal material [39]. On the other hand, the *P. nigra* variants (PND US, PND M) were compared with the aqueous and acetonic *P. nigra* extracts obtained in a baseline study. It was observed that our extracts presented a lower TTC (8.50 ± 0.55 mg/g for PND US; 6.51 ± 0.96 mg/g for PND M) in comparison with the results of the baseline study (10.6 mg/g for aqueous extracts; 17.1 mg/g for acetonic extracts). This study used highly purified condensed tannins for calibration [40].

As for the differences between the extraction methods, in our study the TPC and TTC was higher for the UAE compared to MAE. However a study conducted on *P. radiata* acetonic bark extracts concluded that the MAE resulted in higher yields of polyphenols and tannins compared to the UAE, but different parameters were used for both methods [41]. The differing results may be due to the longer extraction time for the UAE and the lower microwave power used in our study. This statement is reinforced by other studies in which the yields of phenolic compounds were higher for the UAE method compared to MAE if the compared yields were corresponding to the extraction times set in our study (30 min for UAE and 4 min for MAE), while if we compare the yields corresponding to the same extraction times, the MAE method is superior [42,43].

### 3.3. Volatile Compounds Analysis

The results regarding the qualitative and quantitative analysis of the volatile compounds contained in the tested extracts are recorded in Table 1. The main compounds identified in our extracts were α-pinene and β-pinene, their quantity being way above the other identified compounds content. Mainly, PND M had the highest level of α-pinene and β-pinene, significantly higher than all the other experimental variants. The less abundant volatile compounds were camphene, 3-carene, α-phellandrene, limonene, sabinene, myrcene and tricyclene. The highest content of limonene and sabinene were found in PND M too, while relatively higher quantities of camphene and tricyclene were found in the PSV M variant. 3-carene and α-phellandrene were mainly identified in the PSR M variant. Moreover, the quantitative evaluation of the most important volatile compounds show that the extracts acquired by MAE present a higher amount of compounds compared to the extracts acquired via UAE.

Other studies have shown that the volatile compound content of pine species bark may differ. In a study conducted on oil extractives acquired from the bark and wood of *P. sylvestris*, the only compound that was in common with the experimental variants was α-phellandrene. However, similar compounds were found in the oil extractives of *P. sylvestris’* wood, like α-pinene, camphene, sabinene and limonene [44]. Comparing the results with a second study conducted on *P. sylvestris* bark, limonene was the only compound in common and the major compounds being p-cymene and o-cymene as opposed to our major compound (β-pinene) [45]. In the case of *P. nigra* no data was found about the volatile compounds from the bark, but compounds like α-pinene, β-pinene, camphene, sabinene, myrcene, 3-carene, limonene and α-phellandrene were identified in the twigs of the species [46].

### 3.4. UPLC Analysis

The UPLC analysis of the polyphenolic compounds showed that all extracts contained catechin (Rt = 4.53) and epicatechin (Rt = 5.25). All extracts contained significantly higher concentrations of epicatechin than catechin. The method of extraction used seems to influence the composition of extracts, as can be seen in Table 2. Except for the PND US extract, all the extracts obtained by UAE contained higher concentrations of catechin and epicatechin. As was shown by the determination of total polyphenolic compounds, PSV US contained the highest amount of catechin and epicatechin, while the PND US extract contained the lowest amount of the identified compounds.

The pine bark main constituents are flavan-3-ols. Other studies have shown that the polyphenolic compounds found in pine bark are divided into the monomers catechin and epicatechin and the condensed procyanidins [47].

### 3.5. Antioxidant Activity (AOA)

#### 3.5.1. DPPH Assay

**Figure 2:**
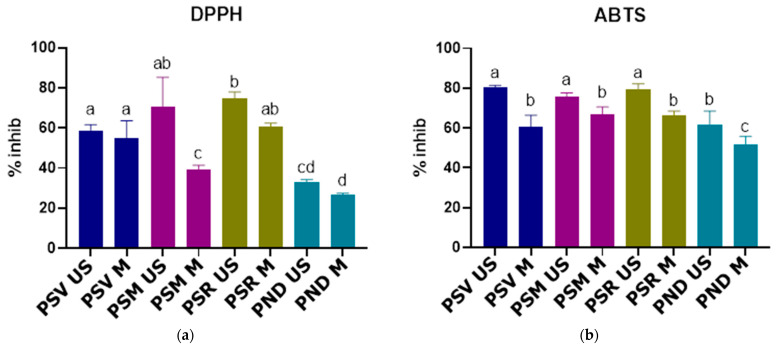
(**a**) Extract antioxidant activity (AOA) expressed by inhibition percentages (DPPH method); (**b**) extract AOA expressed by inhibition percentages (ABTS method); different letters indicate significant differences; PSV US—*P. sylvestris* (Vama) extract obtained by UAE; PSV M—*P. sylvestris* (Vama) extract obtained by MAE; PSM US—*P. sylvestris* (Mediaș) extract obtained by UAE; PSM M—*P. sylvestris* (Mediaș) extract obtained by MAE; PSR US—*P. sylvestris* (Reghin) extract obtained by UAE; PSR M—*P. sylvestris* (Reghin) extract obtained by MAE; PND US—*P. nigra* (Dorohoi) extract obtained by UAE; PND M—*P. nigra* (Dorohoi) extract obtained by MAE.

The highest AOA was exerted by the PSR US variant, having a significant inhibition percentage (74.59% ± 3.35%), in comparison to all the other extracts, excepting PSM US. The PSM US variant presented a significantly higher inhibition percentage (70.36% ± 14.91%) than the other experimental variants, except for PSR M, PSV US and PSR US. Out of all the extracts acquired by MAE, the PSR M variant had the highest inhibition percentage (60.55% ± 1.93%), but the differences were significant only when compared with PND M, PSM M and PND US. The lowest inhibition percentages were registered for the *P. nigra* variants PND US (33.13% ± 1.09%) and PND M (26.66% ± 0.89%) and thus exerting the weakest AOA (Figure 2a).

The inhibition percentages obtained for the experimental variants were compared to the results of three baseline studies. The first study, conducted on methanolic *P. sylvestris* bark extracts indicated a 57.7% inhibition towards the free radicals resulted from the DPPH reagent. This result was inferior compared to the most active experimental variant (PSR US) [23]. The second baseline study analyzed the AOA of *P. sylvestris* and *P. nigra* bark extracts acquired by hot water extraction. A 78.5% inhibition was attained for the *P. sylvestris* extracts, a slightly higher value compared to the best experimental variant (PSR US), but a 87.2% inhibition was obtained for the *P. nigra* extracts, a much higher value compared to the best *P. nigra* variant PND US [26]. A third baseline study focused on the AOA of *P. sylvestris* and *P. nigra* bark extracts acquired by supercritical fluid extraction attained a 58.4% and 46.4% inhibition for the *P. sylvestris* extracts, lower values compared to the most active tested extract. A 53.7% and 52.3% inhibition was obtained for the *P. nigra* extracts, meaning a better AOA compared to the best *P. nigra* variant (PND US) [48]. The inhibition percentages presented in the baseline studies were acquired using the DPPH reagent.

#### 3.5.2. ABTS Assay

In Figure 2b it can be observed that the *P. sylvestris* extracts acquired by UAE, have shown a greater AOA against the ABTS radical, compared to the analogue experimental variants acquired by MAE, PSV US (80.36% ± 1.01%), PSR US (79.34% ± 2.90%) and PSM US (75.78% ± 1.90%) presenting significantly higher inhibition percentages than all the other tested extracts. Even though PND US indicated a significantly lower inhibition percentage (61.91% ± 6.66%) compared to the other extracts acquired by UAE, it was significantly higher than the inhibition percentage of PND M (51.63% ± 4.21%).

Studies regarding the AOA of *P. sylvestris* bark extracts evaluated by the ABTS assay were found, but the results were expressed as mg Trolox equivalents/g vegetal material, thus the results could not be compared with the experimental variants [49,50].

The high antioxidant activity exerted by the extracts might be explained by the presence of catechin and epicatechin in the experimental variants, as both of these compounds were associated in other studies with a high antioxidant capacity [7]. However, the much higher levels of catechin and epicatechin found in the PSV US variant did not lead to a significantly better AOA in the case of ABTS assay and even resulted in a lower AOA in the case of the DPPH assay, when comparing to the other UAE variants. This might indicate that the extracts may contain other vital compounds for the AOA. For example, the presence of galloylated catechins was not assessed by our UPLC analysis, these compounds having a greater antioxidant capacity compared to their simple counterparts, catechin and epicatechin [7].

### 3.6. Antibacterial Activity

The antibacterial activity of the *P. sylvestris* and *P. nigra* bark extracts was evaluated against Gram-positive (*Staphylococcus aureus* ATCC 25923, methicillin resistant *Staphylococcus aureus* ATCC 43300) and Gram-negative (*Escherichia coli* ATCC 25922, *Klebsiella pneumoniae* ATCC 700603 and *Pseudomonas aeruginosa* ATCC 27853) bacteria. All the results are presented in Table 3.

#### 3.6.1. *Staphylococcus aureus* ATCC 25923

All the tested extracts inhibited the growth of this strain, but the PSV US extract was the most efficient, inhibiting the bacterial growth at a concentration of 1.562 mg/mL. The PND M variant had the weakest antibacterial properties against this strain. The extraction method influenced the antibacterial potential of the extracts. The extracts acquired by UAE were superior compared to those acquired by MAE (lower MIC), except for PSM, where MIC was not influenced by the extraction method.

#### 3.6.2. Methicillin Resistant *Staphylococcus aureus* (MRSA) ATCC 43300

It was observed that each extract presented antibacterial activity against this strain, but the most efficient variants were PSV US and PSR US, with a MIC of 1.562 mg/mL. The *P. nigra* variants exerted the lowest antibacterial activity, with MICs of 12.5 mg/mL (PND M) and 6.25 mg/mL (PND US). The UAE method was again more efficient compared to the MAE method.

#### 3.6.3. Gram-Negative Bacteria

In the case of Gram-negative bacterial strains, the antibacterial activity was much lower compared to the Gram-positive antibacterial activity. Out of all the tested extracts, only PSV M and PSV US variants exhibited antibacterial effects at concentrations equal or lower than the maximum concentration tested (100 mg/mL), and only in the case of *E. coli* and *P. aeruginosa*. None of the tested extracts were efficient against the *Klebsiella pneumoniae* ATCC 7,006,003 strain, at concentrations equal or lower than 100 mg/mL.

A baseline study conducted on chloroformic, acetonic and methanolic *P. nigra* bark extracts, evaluated the antibacterial activity of these extracts against different bacteria, including *S. aureus*, *K. pneumoniae*, *E. coli* and *P. aeruginosa* strains. The extracts inhibited the growth and development of all the bacterial strains, except *E. coli*. It must be mentioned that the extracts tested in the baseline study had higher MICs than the maximum limit of our experimental variants [28]. Baseline studies regarding the antibacterial activity of *P. sylvestris* bark extracts on the tested strains were not found. However, a study conducted on extracts acquired from the xylem and phloem of *P. sylvestris* also indicated an inhibiting effect on the growth of MRSA and a reduced, almost absent activity against *E. coli* [51]. Moreover, in previous studies it has been shown that catechin (one of the main components identified in the extracts) has antibacterial properties against *S. aureus* and *E. coli* [52,53] and also catechin, epicatechin and their derivatives have been shown to reduce the resistance of MRSA strains to different antibiotics, thus providing a synergistic effect in combination with several synthetic antibacterial agents [54].

## 4. Conclusions

Taking into account all the results of the present study, it can be concluded that major differences may occur between the chemical compositions and biological effects of the tested pine species, but also between the two extraction methods used. Therefore, it could be noted that the *P. sylvestris* extracts acquired by UAE, presented the highest content of polyphenolic compounds and tannins, while the *P. nigra* variant acquired by MAE, had the biggest amount of α-pinene and β-pinene (main volatile compounds) for the parameters set in the present study. This indicates the potential use of *P. sylvestris* bark as a source of polyphenolic compounds, while *P. nigra* bark could be used as a source of α-pinene and β-pinene. Moreover, it could be observed that the collecting location of the *P. sylvestris* samples influenced their chemical composition and AOA, the samples collected in Vama being the richest in polyphenols and tannins, while samples collected in Reghin resulted in the extracts with the greatest AOA. The high levels of catechin and epicatechin present in the samples collected from Vama, but the relatively small differences in AOA compared to the samples collected from Reghin might suggest that a more complex mixture of compounds is responsible for the AOA of the extracts.

Regarding the biological effects of the bark extracts, the *P. sylvestris* extracts acquired by UAE were superior from an antioxidant and antimicrobial activity standpoint. Even though these extracts showed modest activity against Gram-negative strains, the inhibition percentages of the free radicals and MICs for Gram-positive bacterial strains were better, in comparison to the extracts acquired by MAE and both *P. nigra* variants. This highlights the potential use of these extracts, to reduce oxidative stress and its complications and the treatment possibility of different *S. aureus* infections.

Our results may indicate the importance of capitalizing on these natural wastes, due to their antioxidant and antibacterial activities, but even more benefits could be provided by the high polyphenolic content of these forestry byproducts. Even though the results are promising, further studies are needed to identify and isolate specific bioactive compounds and to assess their mechanism of action in order to have a better understanding of these byproducts and their uses.

## Figures and Tables

**Table 1:** Qualitative and quantitative results of the pine bark extract volatile compounds analysis ± st. dev. (μg/g bark).

Compound	PND US	PSM US	PSR US	PSV US	PND M	PSM M	PSR M	PSV M
α-pinene	2152.84 ± 6.38	621.48 ± 10.04	1272.23 ± 4.25	782.34 ± 3.61	2412.14 ± 6.98	885.40 ± 0.25	1826.10 ± 11.48	1018.04 ± 6.03
β-pinene	3239.12 ± 2.60	3332.87 ± 3.67	3006.23 ± 4.18	1218.91 ± 3.65	3794.11 ± 17.60	3422.53 ± 16.44	3517.65 ± 0.36	1669.02 ± 2.66
Camphene	44.59 ± 2.41	25.18 ± 2.08	55.05 ± 0.30	42.73 ± 1.29	47.77 ± 0.30	39.20 ± 0.16	53.24 ± 0.15	70.14 ± 0.09
3-carene	80.93 ± 0.13	68.25 ± 0.37	75.19 ± 0.23	82.39 ± 0.55	84.17 ± 0.10	68.77 ± 0.26	91.00 ± 0.13	60.27 ± 0.09
α-phellandrene	10.16 ± 0.18	6.38 ± 0.41	9.24 ± 0.21	7.11 ± 0.22	9.75 ± 0.46	7.08 ± 0.04	11.09 ± 0.04	8.00 ± 0.11
Limonene	97.16 ± 0.09	70.31 ± 0.05	88.10 ± 0.04	73.32 ± 0.25	109.90 ± 0.19	74.21 ± 0.05	94.30 ± 0.05	76.67 ± 0.05
Sabinene	49.08 ± 0.08	26.19 ± 0.02	30.04 ± 0.04	19.22 ± 0.03	59.90 ± 0.19	31.34 ± 0.23	37.31 ± 0.05	20.56 ± 0.41
Myrcene	136.31 ± 0.32	112.23 ± 0.23	155.31 ± 0.21	196.96 ± 0.12	139.84 ± 0.29	112.70 ± 0.08	156.20 ± 0.14	198.50 ± 0.46
Tricyclene	9.27 ± 0.09	7.09 ± 0.07	7.18 ± 0.15	22.14 ± 0.06	6.27 ± 0.04	8.19 ± 0.18	7.88 ± 0.07	26.21 ± 0.14

PSV US—*P. sylvestris* (Vama) extract obtained by UAE; PSV M—*P. sylvestris* (Vama) extract obtained by MAE; PSM US—*P. sylvestris* (Mediaș) extract obtained by UAE; PSM M—*P. sylvestris* (Mediaș) extract obtained by MAE; PSR US—*P. sylvestris* (Reghin) extract obtained by UAE; PSR M—*P. sylvestris* (Reghin) extract obtained by MAE; PND US—*P. nigra* (Dorohoi) extract obtained by UAE; PND M—*P. nigra* (Dorohoi) extract obtained by MAE.

**Table 2:** Semiquantitative results of the polyphenolic compounds identified in the pine bark extracts (mg/g bark).

Compound	PND US	PSM US	PSR US	PSV US	PND M	PSM M	PSR M	PSV M
Catechin	0.602	1.289	1.222	8.393	0.588	0.637	1.165	0.958
Epicatechin	2.299	5.005	3.16	37.456	3.817	1.554	3.813	18.779

PSV US—*P. sylvestris* (Vama) extract obtained by UAE; PSV M—*P. sylvestris* (Vama) extract obtained by MAE; PSM US—*P. sylvestris* (Mediaș) extract obtained by UAE; PSM M—*P. sylvestris* (Mediaș) extract obtained by MAE; PSR US—*P. sylvestris* (Reghin) extract obtained by UAE; PSR M—*P. sylvestris* (Reghin) extract obtained by MAE; PND US—*P. nigra* (Dorohoi) extract obtained by UAE; PND M—*P. nigra* (Dorohoi) extract obtained by MAE.

**Table 3:** Minimum inhibitory concentration (MIC) of the tested extracts against the bacterial strains, expressed as mg vegetal material/mL extract.

Tested Bacteria	MIC (mg bark/mL Extract)							
	PSV M	PSV US	PSR M	PSR US	PSM M	PSM US	PND M	PND US
*Staphyllococcus aureus* ATCC 25923	3.125	1.562	6.25	3.125	6.25	6.25	12.5	6.25
Methicillin resistant *Staphyllococcus aureus* (MRSA) ATCC 43300	3.125	1.562	3.125	1.562	6.25	3.125	12.5	6.25
*Escherichia coli* ATCC 25922	100	50	>100	>50	>100	>50	>100	>50
*Klebsiella pneumoniae* ATCC 700603	>100	>50	>100	>50	>100	>50	>100	>50
*Pseudomonas aeruginosa* ATCC 2753	100	25	>100	>50	>100	>50	>100	>50

PSV US—*P. sylvestris* (Vama) extract obtained by UAE; PSV M—*P. sylvestris* (Vama) extract obtained by MAE; PSM US—*P. sylvestris* (Mediaș) extract obtained by UAE; PSM M—*P. sylvestris* (Mediaș) extract obtained by MAE; PSR US—*P. sylvestris* (Reghin) extract obtained by UAE; PSR M—*P. sylvestris* (Reghin) extract obtained by MAE; PND US—*P. nigra* (Dorohoi) extract obtained by UAE; PND M—*P. nigra* (Dorohoi) extract obtained by MAE.

## Data Availability

Data sharing not applicable.
